# A bulk milk tank study to detect evidence of spread of Schmallenberg virus infection in the south-west of Ireland in 2013

**DOI:** 10.1186/2046-0481-67-11

**Published:** 2014-05-27

**Authors:** Alan Johnson, Bernard Bradshaw, Catherine Boland, Padraig Ross

**Affiliations:** 1 Regional Veterinary Laboratory, Department of Agriculture, Food and the Marine, Knockalisheen, Limerick, Ireland; 2 Veterinary Research Laboratory, Department of Agriculture, Food and the Marine, Backweston, Celbridge, County Kildare, Ireland

**Keywords:** Schmallenberg virus, Dairy herds, Bulk tank milk, No evidence of spread

## Abstract

**Background:**

Schmallenberg virus (SBV) was first detected in Germany in November 2011. Confirmation of infection in Ireland was reported on October 30^th^ 2012. The results of a national serological survey carried out in early 2013 suggested that the first introduction of SBV into Ireland probably occurred in the south or southeast of Ireland in the spring or summer of 2012, with subsequent spread eastwards and northwards. It was unclear at that stage whether the virus had survived the winter period and would continue to spread in 2013. The purpose of this study was to monitor the spread of the virus in the mid-west region through the summer and autumn of 2013 using bulk tank milk from selected dairy herds.

**Findings:**

Seventy two dairy farmers were recruited to participate in the bulk milk tank study. Each farmer agreed to collect a bulk tank milk sample on a weekly basis from early June. A total of 988 samples were received between June 5^th^ and December 3^rd^ 2013 and these were analysed using an indirect ELISA test. Of the initial set of 72 samples received between June 5^th^ and July 16^th^, nine were positive, one was inconclusive and 62 were negative. By the end of the study in early December 2013 only one new farm turned positive. This was the farm that had initially tested inconclusive.

**Conclusion:**

The study results suggest that the anticipated spread of SBV across Ireland from the south and south-east did not occur during 2013.

## Findings

Schmallenberg virus (a previously unknown *Orthobunyavirus*) was first detected in North Rhine-Westphalia, Germany in November 2011 (Hoffmann and others, [1]). Infection with the virus has been associated principally with congenital malformations in calves, lambs and kid goats. It is believed that the virus was not present in Europe before 2011, but that before the end of that year the infection had spread over a large area of Western Europe, including Great Britain (Beer and others, [2]). Some species of *Culicoides* biting midges have been shown to be capable of harbouring high levels of Schmallenberg virus (SBV), and the evidence is strong that they are natural vectors linked with its spread (Elbers and others, [3]). Evidence of SBV infection in Ireland was first reported on October 30^th^ 2012 (Bradshaw and others, [4]) following positive test results on bovine foetal material sent to the Department of Agriculture, Food and the Marine’s (DAFM) Regional Veterinary Laboratory (RVL) in Cork from four dairy herds in that county. A real-time quantitative reverse transcription polymerase chain reaction (RT-qPCR) test, developed at the Friedrich-Loeffler-Institut in Germany (Bilk and others, [5]), has been used to detect viral genome on 49 cattle and 30 sheep farms from eleven counties in the southern part of Ireland (Cork, Waterford, Wexford, Kilkenny, Wicklow, Dublin, Laois, Carlow, Tipperary, Limerick and Kerry) between then and the end of 2013.

A serological survey was carried out during the winter of 2012 and spring of 2013 to better understand the distribution of exposure to the virus (Bradshaw, personal communication) in the Republic of Ireland during 2012. Six animals were tested from a minimum of 17 herds in each county for evidence SBV antibodies using an indirect enzyme-linked immunosorbent assay (ELISA). The results showed that counties in the south and southeast had the highest herd seroprevalence (>50%) and those in the north and northwest having the lowest herd seroprevalence (0%-10%). The results of the serological survey and RVL surveillance suggested that the first introduction of SBV into Ireland probably occurred into the south or southeast of Ireland in the spring or summer of 2012, with subsequent spread eastwards and northwards in the direction of the prevailing wind. It was unclear whether the virus had survived the following winter period or continued to spread in early 2013.

A vaccine (Bovilis SBV, MSD Animal Health), developed primarily to prevent foetal infection, has been licensed for use in the United Kingdom since May 2013, and in the Republic of Ireland since June 2013. It is the first and only SBV vaccine to come to the market to date. Once vaccination has been carried out in a herd it is not possible to differentiate between vaccinated and naturally infected animals using antibody detection tests.

Limerick RVL, one of six RVLs in the Republic of Ireland, carries out a surveillance role for DAFM. It receives submissions mainly from the surrounding counties of Clare, Tipperary, Limerick and Kerry. In 2013, ovine and bovine foetuses and stillbirths presented to the RVL with congenital malformations were tested for SBV. Of the 56 bovine and 9 ovine samples tested during 2013 by Limerick RVL, only two bovine foetuses (1 in Kerry and 1 in Limerick) were found to be positive for SBV using the qRT-PCR test.

**Figure 1:**
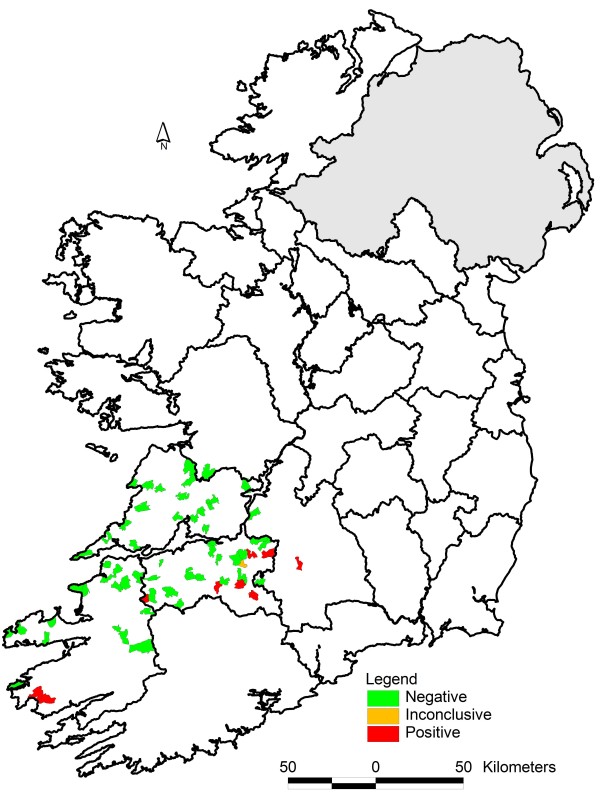
The District Electoral Division location and result status of the 72 dairy farms involved in the Schmallenberg Virus Bulk Milk Tank Longitudinal Survey during the summer and autumn of 2013.

In early summer 2013 a longitudinal study of dairy farms in the catchment area of Limerick RVL was initiated to monitor the expected spread of SBV across the country in summer and autumn. It was decided to use an indirect ELISA (ID Screen Schmallenberg virus Milk Indirect; ID.vet; Montpellier, France) for the detection of SBV antibodies in bulk tank milk as it had been reported to be a useful method for detecting herd level exposure to SBV (Humphries, [6]; Tarlinton and others, [7]). A geographically diverse group of veterinary practitioners was asked to nominate dairy farmers from their practices to participate in the study. Seventy two dairy farmers were recruited from sixty six separate district electoral divisions (DEDs) in the counties of Clare, Galway, Kerry, Limerick and Tipperary (Figure 1). None of the farmers selected were using or planned to use the licensed SBV vaccine during the course of the study. Each farmer agreed to collect and submit a bulk tank milk sample on a weekly basis from early June. Milk collection bottles preloaded with potassium dichromate preservative tablets (Lactabs Mark III, Thompson and Capper Ltd.) and pre-addressed envelopes were supplied to each of the participating farmers.

The initial set of 72 samples was received between June 5^th^ and July 16^th^. Nine of the samples were positive (S/P > 40%), one was inconclusive (30% < S/P < =40%) and 62 were negative (S/P < =30%). The positive samples were located in south Kerry, east Limerick and Tipperary (Figure 1), locations close to the counties of Cork and Tipperary, which had been shown to have a high percentage of SBV antibody positive holdings (Bradshaw, personal communication).

A total of 988 samples were received between June 5^th^ and December 3^rd^ 2013. The number of samples per farmer ranged from two to 24. Where positive test results were obtained on two or more consecutive samples from any one farm it was considered that the farm was exposed to SBV and no further information was required for the purposes of this survey.

As the sampling and testing continued through the summer and autumn months, the number of positive herds rose only by one, to ten, suggesting that the anticipated spread of the virus across the survey area had not occurred. The one herd that turned positive had initially had an inconclusive test result.

Although not definitive on its own, this longitudinal study suggests that the anticipated spread of SBV across the country of Ireland via infected midges did not occur during 2013, despite the presence of a large number of non-infected hosts. Further studies need to be carried out to confirm this and to establish why the anticipated spread did not occur. It is possible that the weather conditions prevailing during the winter of 2012/13 were not conducive to the survival of the midge species capable of acting as vectors of SBV, though previous studies have shown that transmission of SBV is possible, even during central Europe’s winter conditions, when minimum temperatures rise above a certain threshold for several days (Wernike and others, [8]). Many of the midge species identified as highly probable vectors of SBV (Balenghien and others, [9]) have been identified previously in Ireland (McCarthy and others, [10]). Ireland has a milder winter climate than central European countries, and monthly average temperatures rarely fall below 6.0 degrees Celsius (°C). However, in the months of February and March 2013, the monthly average temperature at Shannon airport (located in the south-west of Ireland) fell well below the mean for the years 1981 to 2010, averaging 5.6°C in February and 4.7°C in March (Met Éireann, [11]). These weather conditions may have assisted in reducing the number of viable vector midges, thus preventing the infection from spreading significantly further from those farms infected in 2012.

## Abbreviations

SBV: Schmallenberg virus; DAFM: Department of Agriculture, Food and the Marine; RVL: Regional Veterinary Laboratory; qRT-PCR: Quantitative reverse transcriptase polymerase chain reaction; ELISA: Enzyme-linked immunosorbent assay; S/P: Sample to positive ratio; °C: Degrees celsius.

## Competing interests

The authors declare that they have no competing interests.

## Authors’ contributions

AJ conceived the study, participated in its design and co-ordination and drafted the manuscript. BB participated in the design of the study and helped draft the manuscript. CB carried out the sample preparation and testing. PR carried out the sample preparation and testing. All authors read and approved the final manuscript.

## Authors’ information

AJ is a senior research officer based in the Department of Agriculture, Food and the Marine’s Regional Veterinary Laboratory in Limerick.

BB is a senior research officer based in the virology division of the Department of Agriculture, Food and the Marine’s Central Veterinary Research Laboratory, Backweston, Celbridge, Co. Kildare.

CB and PR are laboratory analysts based in Department of Agriculture, Food and the Marine’s Central Veterinary Research Laboratory, Backweston, Celbridge, Co. Kildare.
